# Clinical outcome of various limb salvage surgeries in osteosarcoma around knee: Megaprosthesis, extracorporeal irradiation and resection arthrodesis

**DOI:** 10.1016/j.amsu.2019.04.005

**Published:** 2019-04-24

**Authors:** Achmad Fauzi Kamal, Primadika Rubiansyah

**Affiliations:** Department of Orthopaedic and Traumatology Dr.Cipto Mangunkusumo General Hospital/Faculty of Medicine Universitas Indonesia, Jakarta, Indonesia

**Keywords:** Limb salvage surgery, Megaprothesis, Extracorporeal irradiation, Arthrodesis

## Abstract

**Background:**

We evaluated the outcome and the survival rate of Limb salvage surgeries (LSSs) in osteosarcoma around knee by using megaprosthesis, ECI autograft, and modified arthrodesis of the knee with metallic plus bone cement (MAMC).

**Methods:**

We reviewed 35 cases of osteosarcoma around the knee that was treated by megaprosthesis, ECI autograft and MAMC from 2012 to 2017. The survival, local recurrence, metastases, complications and functional MSTS score were evaluated for each operation technique. Kaplan-Meier was used to describe the survival rate for each technique.

**Result:**

Megaprostheses group had an excellent MSTS score (78.7%), the ECI group (72.3%) and MAMC group (68.4%). Local recurrence occurred in the megaprothesis group (0%), the ECI group (9.1%) and MAMC group (20%). Infection occurred in 3 cases of ECI (13.6%) while only 2 (40%) cases in MAMC group and 1 case (12.5%) in the megaprostheses group. Aseptic loosening occurred in the megaprostheses group 1 case (12.5%) and MAMC 1 case (20%). Metastases occurred in 18.2% of the ECI group compared to 25% of the megaprostheses group and 40% of the MAMC group. The megaprosthesis group had an overall survival rate of 90.9 months, whilst the ECI group is on 94.6 months and the MAMC group was 47.2 months.

**Conclusion:**

Megaprosthesis showed good-excellent functional outcome and survival rate. ECI that is an option in LSS has good functional outcome as well. Knee arthrodesis with MAMC it is still an option to perform LSS even in the advanced local stage of the disease.

## Highlights

• Various reconstruction options are available for the reconstruction of the distal femur and proximal tibia osteosarcoma.
• Megaprosthesis showed good-excellent functional outcome and survival rate.
• ECI that is an option in LSS has good functional outcome as well.
• Knee arthrodesis with MAMC it is still an option to perform LSS even in the advanced local stage of the disease.

## 1 Background

The incidence of osteosarcoma in all populations is approximately 4–5 per 1,000,000 population. It is higher in adolescents to 8–11 per one million population per year at the age of 15–19 years [[1], [2], [3], [4]]. Overall, osteosarcoma has a moderate incidence rate, with 10–26 per million new cases worldwide each year [2,4].

The concept of limb salvage surgery (LSS) has gradually developed over the last twenty-five years. These advancements in bone tumor management have given both surgeons and patients more options for treatment, other than mere limb ablation. Currently, 90–95% of patients with sarcoma of the extremities that were administered in tertiary referral centre can undergo musculoskeletal LSS with a successful result [[5], [6], [7], [8], [9]].

The main aim of reconstructive surgery after oncologic resection include providing skeletal stability, adequate wound coverage to allow subsequent adjuvant therapy, restoration of acceptable functional capability and desirable aesthetic outcome when possible. Reconstruction of the bone defect after completion of the tumor resection depends on the surgeon experience and available resources in the institution [10].

Currently, we are able to choose from a variety of methods of reconstruction, including os-teoarticular allografts/autografts, intercalary allografts/autografts, autograft/allograft-prosthetic composites, arthrodesis with autogenous or allogenic bone, custom-made prostheses, and rotationalplasty [7,[11], [12], [13], [14]]. In this study, we analyzed the outcome and survival rate of osteosarcoma patients that underwent LSS by using megaprosthesis, extracorporeally irradiated (ECI) autograft, and modified arthrodesis of the knee with metallic plus bone cement (MAMC) in our hospital, in Jakarta, Indonesia.

## 2 Methods

We reviewed osteosarcoma data from musculoskeletal oncology registries, medical records, and follow-up care in an outpatient clinic treated in our hospital from 2012 to 2017. All lesions were clinically, radiologically, and histologically confirmed as osteosarcoma. The criteria for LSS were no major neurovascular involvement of the tumor, which evaluated preoperative through MRI, no local infection and adequate soft tissue coverage. A patient who was supported by universal health coverage from our government or could pay megaprosthesis could undergo LSS with megaprosthesis. ECI autograft was indicated for an osteosarcoma patient who had a good bone stock (blastic lesion) as an alternative of the limb salvage procedure regarding limitation of megaprostheses. MAMC was indicated for the patient who had much more soft tissue extension without neurovascular bundle involvement or for the patient who refused amputation.

**Fig. 1:**
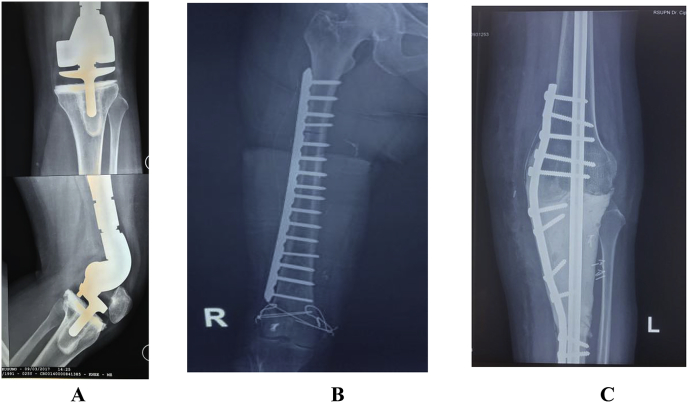
Post -operative radiographs of patients with osteosarcoma around the knee who underwent LSS: A). Megaprosthesis; B). ECI autograft and C). MAMC.

Age was classified into groups of decades. Tumor size was divided into 2 groups (<8 cm and >8 cm). Types of patient management were divided into LSS using megaprosthesis, ECI, and MAMC of the knee (Fig. 1). The patients who did not complete the profile data and the follow-up of their condition were excluded. Local recurrence, metastases, complications of LSS, survival, and functional score were evaluated.

Local recurrence was detected by physical, radiologic, and histopathology examinations. Me-tastasis was confirmed by chest X-ray or computed tomography scan, and/or bone scintigraphy. We observed the complications such as infection and loosening of the implant. Survival was measured since patients underwent the LSS until the time of follow up or death. Functional status was evaluated by the Musculoskeletal Tumor Society Scoring System (MSTS). Kaplan-Meier curve was used to describe the survival analysis, its correlation with types of surgery.

The study was reported in line with the STROCSS criteria [15]. And register in open access database (UIN: researchregistry4576).

## 3 Results

From 2012 to 2017 period, there were 35 consecutive osteosarcoma patients who underwent LSS. Patient characteristics are given in Table 1. The mean follow up was 58.7 months. Twenty-six (57.6%) patients were in young age group (11–20 year-old) with a median of 16.2 year-old (interquartile range 9.0–29.0 year-old). Male and female patients were 23 (65.7%) and 12 (34.3%) respectively. There were the elevation of lactic dehidrogenase level and serum alkaline phosphatase level pre-operatively in 29 patients (83.7%).

**Table 1:** Patients characteristics.

Variable	Total
Male, n (%)	23 (65.7%)
Female, n (%)	12 (34.3%)
Age	16.2 ± 3.47
Mean LDH	
Pre Operation	1028.3 ± 1810,14
Post Operation	430.5
Mean ALP	
Pre Operation	237.7 ± 202,06
Post Operation	105.4
Mean Follow up Period	58.7 ± 23.29
HUVOS Grade	
I-II n (%)	16 (45.71%)
III-IV n (%)	19 (54.28%)
Enneking Stage	
IIB, n (%)	31 (88,57%)
III, n (%)	4 (11.43%)
Tumor Size	
<8 cm	14 (40%)
≥8 cm	21 (60%)
Location	
DF, n (%)	21 (60%)
PT, n (%)	14 (40%)
Mean MSTS Score	72.1 ± 5.13
Infection	
Yes, n (%)	6 (17.1%)
No, n (%)	29 (82.9%)
Recurrence	
Yes, n (%)	3 (8.6%)
No, n (%)	32 (91.4%)
Aseptic Loosening	
Yes, n (%)	2 (5.7%)
No, n (%)	33 (94.3%)
Metastatic	
Yes, n (%)	8 (22.9%)
No, n (%)	27 (77.1%)

**Hb,** Hemoglobin; **Ca,** Calcium; **DF,** Distal Femur; **PT,** Proximal Tibia; **LDH,** Lactate Dehydrogenase; **ALP,** Alkaline Phosphatase.

Most of the patients came in Enneking stage IIB (88.57%) while 60% of patients came with tumor size more than 8 cm. Four patients (11.43%) came in Enneking stage III. Nineteen patients (54.28%) had a good response to chemotherapy as Huvos grade III - IV.

**Fig. 2:**
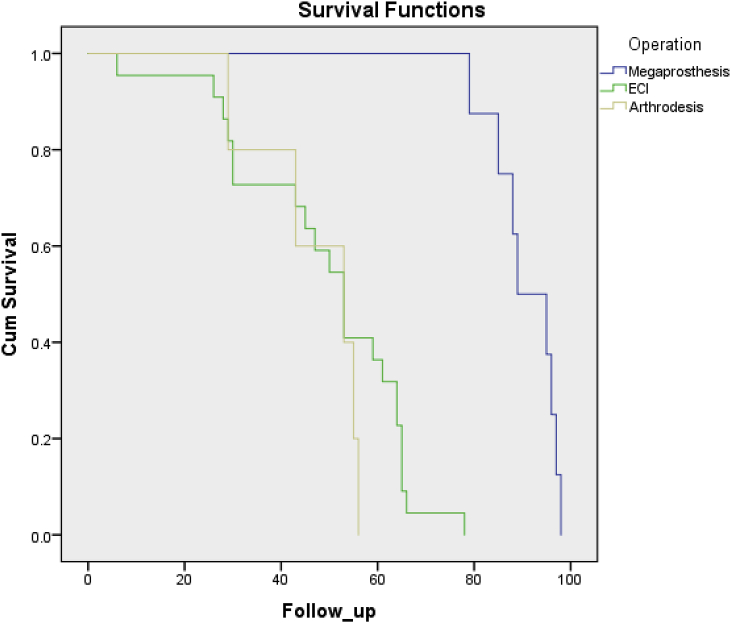
Kaplan-Meier curve showed the survival analysis of the osteosarcoma patients who underwent LSS with megaprosthesis, ECI autograft, and MAMC.

The average of MSTS score for all groups was 72.1 in 1 year follow up. Megaprosthesis group had an excellent MSTS score (78.7%) compared to the ECI group (72.3%) and MAMC group (68.4%). Local recurrence occurred in megaprosthesis group 0 (0%), ECI group 2 of 22 (9.1%) and MAMC group 1 of 5 (20%). Six patients (17.1%) had a post-operative infection: a case in megaprosthesis group (12.5%), 3 cases in the ECI group (13.6%), and 2 cases in MAMC group (40%). Aseptic loosening occurred in the megaprosthesis group (12.5%) and MAMC (20%). Three patients (8.6%) had local recurrence and 8 patients (22.9%) had metastases during follow up. Metastases occurred in 18.18%, 25% and 40% in ECI, megaprostheses and MAMC group respectively (Table 2). The megaprosthesis group had an overall survival rate of 90.9 months, whilst the ECI group is on 94.6 months and the MAMC group was 47.2 months (Fig. 2).

**Table 2:** Comparison of the outcome of LSS with megaprosthesis, ECI autograft, and MAMC.

	Megaprostheses (n = 8)	ECI (n = 22)	MAMC of Knee (n = 5)
MSTS Score	78.7% (71.2%–83.3%)	72.32% (64.2%–76.6%)	68.36% (61.3%–70.12%)
Local recurrence	0% (0)	9.1% (2)	20% (1)
Infection	12.5% (1)	13.6% (3)	40% (2)
Aseptic Loosening	12.5% (1)	0% (0)	20% (1)
Metastases	25% (2)	18.2% (4)	40% (2)

## 4 Discussion

Conventional osteosarcoma is more common in men than women by a ratio of 3:2. The tumor most commonly affects patients within the 2nd decade of life and more than 60% in patients less than 25 years old [1,3,16]. Most osteosarcoma patients are young. Therefore, the treatments are supposed to preserve the limb and to maintain function without major complications orrecurrences over the long-term [17].

In the present study, the male and female ratio is 1.92 : 1 with a peak incidence in the second decade of life. Kamal et al. [1] reported male and female ratio is 1.54 : 1 for all osteosarcoma. Our result is similar with other study reported by Picci et al. [18] that osteosarcoma is more common in male patients, mostly in the second and third decade of life.

Our study demonstrated that most patients came in Enneking stage IIB (88.57%). It was comparable with Huang et al. [13] study. They reported 89.4% osteosarcoma cases came in Enneking stage IIB. In our study, 54.71% patients had a good response to chemotherapy (Hu-vos III and IV). That result was comparable with Puri et al. [19] study who reported 20% of osteosarcoma cases had Huvos IV and 35% had Huvos III. Those studies show better result than our previous study that reported only 40.7% patients with Huvos III and IV [1]. The patients included in these study are all covered by the national health insurance, therefore, the majority of them had already been undergoing scheduled chemotherapy.

Osteosarcomas are commonly seen around the knee, especially at the distal end of the femur. Being a weight bearing bone, reconstruction of the distal femur and proximal tibia following excision of the tumors is of utmost importance. With modern day effective treatment using neo-adjuvant and adjuvant chemotherapy, LSS is the recommended treatment for osteosarcomas wherever possible.

Various reconstruction options are available for the reconstruction of the distal femur and proximal tibia following tumor excision that includes megaprosthesis, ECI autograft and MAMC. In developed countries, because of the accessibility and ease of insertion, megaprosthesis are commonly being used. The megaprostheses of for early mobility with the maintenance of joint motion in these cases [20].

Many studies mentioned that LSS is an accepted treatment for tumors around the knee. In the developed countries, megaprosthesis is the method of choice to restore function and results in optimal patient satisfaction [[21], [22], [23], [24], [25], [26]]. The advantages of megaprostheses around knee reconstruction allows the patients to do immediate weight bearing, maintenance of joint mobility, and early return to activities. Functional outcomes after megaprosthesis reconstruction were generally good to excellent daily living activities [21,23,24,26]. In this study, our patients with post-operative megaprosthesis demonstrated good functional outcome with good to excellent MSTS score (mean score 78.7%).

Megaprosthesis reconstruction gives the most favorable clinical result in terms of functional outcome and complication rates. However, megaprosthesis has the limitation of long term survival of prosthesis and high cost implant including Indonesia or many other developing countries. In Indonesia, reconstruction with megaprosthesis has started since 2011. Fortunately, the osteosarcoma patients (and other bone tumors) indicated for megaprosthesis were supported by our hospital and universal health coverage from the Indonesian government.

In our study, 12.5% of the cases had an infection that needed serial debridement and prosthesis removal. There was also one case that had aseptic loosening. Sharil et al. reported good functional outcome with an infection rate of 12.86%. He also reported one case with mechanical failure [21]. Tunn et al. [27] reported good functional outcome using MSTS and TESS score. These reconstructions were also insufficient due to the lack of muscle strength and subsequent instability of the adjoining joint leading to impaired function. On the other hand, infection and loosening have remained as the main issues following reconstruction with the megaprosthesis [20].

Most patients in developing countries could not afford the megaprosthesis reconstruction. Thus, they are treated with other techniques. Biological reconstructions and arthrodeses are considered an alternative treatment for patients who cannot afford megaprosthesis. Recycling of the resected segment ECI is one type of biological reconstruction [17].

From a developing nation's perspective, reimplantation of ECI tumor bearing bone segments is an appealing option. It allows immediate and anatomical correct filling of the defect [28]. Regarding previous literature, It has an effect in killing the tumor cells [17]. ECI has several potential advantages. ECI autograft is a useful alternative of LSS regarding limitation of megaprosthesis and allograft. Bone stock can be maintained and suitable for the patient and can preserve epiphyseal plate in the immature patient [29,30]. In our study, the ECI group showed good functional outcome with MSTS score 72.32%.

Delayed weight bearing is the main problem in the ECI group. Therefore, in the short-term follow-up, ECI showed lower MSTS score than megaprosthesis, but in long-term follow-up, it was closely similar. In the present study, ECI showed infection rate 13.6% and 9.1% cases of local recurrence which need amputation. Our previous study ECI demonstrated a good MSTS score (70.63%) with 20% infection rate. Local recurrence also reported as high as 20% [7]. Sharma et al. [31] reported an infection rate of 14%. Infection can result into delayed/non‐union of the bone graft, failure of the graft, and delay in the subsequent course of chemotherapy and therefore every effort should be done to minimize the infection [30].

Many studies reported that local recurrences are correlated with the quality of surgical margins and response to chemotherapy [32,33]. Bacci et al. [32] reported that local recurrence did not correlate with patients' age and sex, histologic subtype, site and tumor volume, presence of pathologic fracture, chemotherapy regimen and type of surgery.

Megaprosthesis replacement has been the preferred technique for treating osteosarcomas around the knee joint. However, functional mobile knee reconstruction requires active knee extension. When the quadriceps must be resected with the tumor, the extensor mechanism should be reconstructed or the patient should have knee arthrodesis [22]. An arthrodesis may also be indicated as a salvage or back-up procedure after a prosthetic replacement or an osteoarticular allograft has failed [13]. The treatment of malignant or aggressive tumorsat the knee by resection-arthrodesis has been used for many years. Merle d’Aubigne's modification of Juvara's resection-arthrodesis is relatively simple and has few complications. Wide resection, stable fixation byan intramedullary rod, correct alignment and sufficient auto and allogenic bone grafts are the main requirements for a successful outcome [34].

Enneking and Shirly reported 20 cases of local resection and arthrodesis employing an intra-medullary nail and autogenous segmental cortical grafts obtained from the same extremity. The indication for selection of the procedure was a lesion in the epiphyseal region of the femur or tibia in such a way that adequate resection with preservation of joint function was not possible. They continued with external support for a period of one year if union occurred. They claimed 95% good functional results at the end of two years from surgery by this method. Four patients had a nonunion while four patients had spontaneous fatigue fractures of their grafts. All sites of nonunion subsequently healed four to 11 months after supplementary iliac bone grafting [35].

We modified resection-arthrodesis with a simpler and cheaper method MAMC. Knee arthrodesis MAMCis an option of LSS techniques in patients with extensor apparatus deficiency, extensive soft tissue involvement by the tumor, no availability of high-cost megaprosthesis, unable to use ECI autograft or limited availability of massive allograft. MAMC is also viewed as a salvage procedure which may serve to avoid amputation. In our study, we combined Kuncher nail, bone cement and plate screws as implant devices.

Knee arthrodesis was originally proposed to restore limb function, particularly in young patients with partial or complete loss of their extensor apparatus. An immovable knee in good alignment and position is considered to be an appropriate sacrifice in order to achieve a stable, pain-free limb [22]. Patients who received an arthrodesis had a more-stable limb and performed the most-demanding physical work, but they had difficulty in sitting [36].

For the arthrodesis patients, the local recurrence rate was expected to be high due to the extensive involvement by tumors or contamination due to previous surgery. However, the local recurrence rate was not as high as expected because a generously wide surgical margin without compromise could be achieved when performing resection arthrodesis [22]. Our study demonstrated one patient (20%) had a local recurrence and two patients (40%) had an infection that needed debridement. Shih et al. [22] reported the local recurrence rate was acceptable for the limb salvage procedures using knee arthrodesis (11.1%) and infection rate 8.3%.

Another previous study by Kamal et al. [1,7] reported a five-year survival rate of 54.97 ± 9.8% for LSS using ECI, while Sharma et al. [31] affirmatively reported a survival rate as high as 64% for the same surgical technique. Patients with tumor size < 8 cm in diameter with a good type of Huvos always had a better survival rate than those with tumor size > 8 cm in diameter with a poor type of Huvos [1,37]. It explained that LSS using knee arthrodesis had a lower survival rate than megaprosthesis and ECI. Furthermore, this is backed by a study of Shih et al. 21, who reported the survival rate of LSS using knee arthrodesis being 39%, which is significantly lower than the 60% of five-year survival rate of patients with endoprosthesis. This reflects the fact that the patients whom are indicated for arthrodesis are usually in the advanced stage of the disease. As a retrospective study, this research is designed to analyse our pre-existing data which is subject to potential bias as a result retrospective study (data collection from the medical records of patients). This study is not randomized, with different number of patients in each group. To reduce a potential selection bias, we determined the criteria for LSS in all groups. A patient who was a minimal age 14 years and indicated for LSS could undergo any types of reconstruction. In addition, ECI autograft was indicated for a younger patient who had a good bone stock as an alternative of the limb salvage procedure. However, MAMC was indicated for the patient who had much more soft tissue extension without neurovascular bundle involvement or for the patient who refused amputation. In other words, the last group is not comparable to other groups.

## 5 Conclusion

Various reconstruction options are available for the reconstruction of the distal femur and proximal tibia following tumor excision that includes megaprosthesis, ECI autograft and MAMC. Reconstruction of the bone defect after completion of the tumor resection depends on available resources in the institution. For the patients in developing countries may not afford the megaprosthesis reconstruction, they could be treated with other techniques such as ECI autograft, MAMC or other biological reconstruction methods. Megaprosthesis showed good-excellent functional outcome and survival rate. ECI that is an option in LSS has good functional outcome as well. Knee arthrodesis with MAMC it is still an option to perform LSS even in the advanced local stage of the disease.

## Ethical approval

Ethical approval no 537/UN.F1/ETIK/2019.

From Faculty of Medicine Universitas Indonesia.

## Sources of funding

The authors declare that sponsors had no such involvement.

## Author contribution

AFK contributed to performed the operation, data collection, analysis and interpretation, manuscript drafting, revising, and approval for publishing; PR contributed to assist the operation, data collection, analysis and interpretation, manuscript drafting, revising, and approval for publishing.

## Conflicts of interest

The authors declare that there is no conflict of interests regarding the publication of this paper.

## Research registration number

Researchregistry4576.

## Guarantor

Guarantor in this study is AFK.

## Provenance and peer review

Not commissioned externally peer reviewed.

## Consent

The patient received an explanation of the procedures and possible risks of the surgery, and gave written informed consent.
